# Seroreactivity against Specific L5P Antigen from *Mycobacterium avium* subsp. *paratuberculosis* in Children at Risk for T1D

**DOI:** 10.1371/journal.pone.0157962

**Published:** 2016-06-23

**Authors:** Magdalena Niegowska, Novella Rapini, Frank Biet, Simona Piccinini, Sylvie Bay, Roberta Lidano, Maria Luisa Manca Bitti, Leonardo A. Sechi

**Affiliations:** 1 Department of Biomedical Sciences, University of Sassari, 07100, Sassari, Italy; 2 Pediatric Diabetology Unit, Policlinico di Tor Vergata, University of Rome Tor Vergata, 00133, Rome, Italy; 3 UMR1282, Infectiologie et Santé Publique (ISP-311), INRA Centre Val de Loire, F-37380, Nouzilly, France; 4 Institut Pasteur, Unité de Chimie des Biomolécules, Département de Biologie Structurale et Chimie, Paris, France; Indian Institute of Technology Delhi, INDIA

## Abstract

**Aims/Hypothesis:**

Although numerous environmental agents have been investigated over the years as possible triggers of type 1 diabetes (T1D), its causes remain unclear. We have already demonstrated an increased prevalence of antibodies against peptides derived from *Mycobacterium avuim* subsp. *paratuberculosis* (MAP) homologous to human zinc transporter 8 protein (ZnT8) and proinsulin in Italian subjects at risk for or affected by T1D. In this study, we compared titers of the previously detected antibodies with seroreactivity to MAP lipopentapetide (L5P) that recently emerged as a strong immunogenic component able to specifically distinguish MAP from other mycobacteria.

**Methods:**

Plasma of 32 children and youth at risk for T1D including follow-up samples and 42 age-matched healthy controls (HC) recruited at the Tor Vergata University Hospital in Rome was analyzed by indirect ELISA for the presence of antibodies against MAP-derived epitopes MAP3865c_133–141_, MAP3865c_125-133_, MAP2404c_70-85_ and MAP1,4αgbp_157-173_ along with their ZnT8 and proinsulin homologs. The data were analyzed through two-tailed Mann-Whitney *U* test and relation between variables was determined by principal component analysis.

**Results:**

Responses to L5P were not detectable in subjects whose initial seroreactivity to MAP peptides and their human homologs was lost in follow-up samples, whereas anti-L5P antibodies appeared constantly in individuals with a stable immunity against MAP antigens. The overall coincidence in positivity to L5P and the four MAP epitopes both in children at risk for T1D and HC exceeded 90%.

**Conclusions:**

MAP-derived homologs may cross-react with ZnT8 and proinsulin peptides inducing immune responses at a young age in subjects predisposed for T1D. Thus, L5P may have a diagnostic value to immediately indicate the presence of anti-MAP seroreactivity when evaluation of a more complex antibody status is not required. Almost complete coincidence in responses to both types of antigens lends support to the involvement of MAP in T1D.

## Introduction

Type 1 diabetes (T1D) is an autoimmune disease of unknown origin clinically developing in children and youth. Over the years numerous studies have described possible causative factors that combine genetic predisposition and environmental agents, however, thus far no clear evidence regarding the most probable trigger responsible for a cascade of events leading to β-cell immunity has been provided. In the previous reports, we portrayed the association of *Mycobacterium avium* subsp. *paratuberculosis* (MAP) with T1D in adults and children from mainland Italy and Sardinia [1, 2, 3, 4], the latter characterized by the second highest T1D incidence worldwide. MAP raised our interest due to its noticeable prevalence in livestock herds causing chronic intestinal inflammation, namely Johne’s disease, and detection in dairy products including infant milk formula [5, 6] that has been object of concerns as a source of exogenous proteins putatively contributing to T1D development [7]. MAP is shed to the environment in milk and faeces of infected animals with subsequent risk of daily exposure; evidences of its resistance to commercial pasteurization process [8, 9] along with cases of isolation from human breast milk [10] suggest a straightforward transmission pathway. Moreover, involvement of MAP in the pathogenesis of other autoimmune diseases such as Hashimoto’s thyroiditis [11] has been hypothesized in addition to the broadly assessed link with Crohn’s disease, characterized by symptom similarity with Johne’s disease.

Due to a very slow growth, a possible role of MAP in several human disorders has been investigated by PCR amplification of specific gene sequences and the presence of antibodies (Abs) directed against numerous MAP-derived antigens. The surface glycopeptidolipids (GPLs) are known to interfere with host’s immune system and, despite the lack of strain specificity, constitute the major antigenic component for diagnosis of mycobacterial infections. They are among the main free glycolipid elements of the outer membrane peculiar to several clinically-relevant species belonging to the *Mycobacterium avium* complex [12], including MAP. However, close phylogenetic relationship of MAP and *M*. *avium* raises difficulties for specific immunodetection with possible false-positive reactions resulting from the presence of both strains in the environment. In the last years, analyses of mycobacterial genomes permitted to indentify the production of lipopentapeptide (L5P) in MAP as a distinctive feature among subspecies [13]. Studies in MAP-infected ruminants showed a strong immunogenicity of L5P [14] and, more recently, highly specific IgG responses to L5P were verified in patients affected by Crohn’s disease [15].

Our earlier reports demonstrated that MAP epitopes making part of glucan branching protein (MAP1,4-αgbp), putative regulator for proline utilization (MAP2404c) and two portions of cation efflux membrane protein (MAP3865c) induce high seroreactivity in children at risk for T1D [16]. The selected peptides present sequence homology to human proinsulin (PI) or zinc transporter 8 (ZnT8); Abs against both proteins circulate in blood of individuals with T1D even before manifestation of clinical symptoms and are used to diagnose autoimmune diabetes [17, 18]. The present study aimed to compare seroreactivity against L5P antigen to previous results involving the four MAP-derived peptides and their homologous fragments in the same subjects. Furthermore, occurrence and duration of cross-reactivity due to epitope homology with human T1D autoantigens was evaluated upon validation with synthetic L5P.

## Methods

### Subjects

32 subjects at risk for T1D (n = 19 males and n = 13 females, mean age 8.90±3.52 years) and age-matched healthy volunteers (HC; n = 42, mean age 6.90±3.55 years) were selected in blind from a dataset of children and youth recruited for a previous study [16] upon periodical visits at the Tor Vergata University Hospital of Rome, Italy. Additionally, further time-point samples of 11 T1D at-risk children were included, giving in total 50 samples. T1D risk was verified by the presence of disease familiarity, multiple islet cell autoantibodies and/or high risk HLA genotype. Exclusion criteria for HC were a history of autoimmune disorders and recent inflammatory episode. Plasma separated from whole venous blood samples was analyzed for the presence of Abs against L5P, ZnT8, PI and the homologous MAP-derived peptides. Bioethical Committees of the University of Sassari and the Tor Vergata University Hospital of Rome, Italy, approved the study protocols. Written informed consent was obtained from legal tutors of all study participants

### MAP antigens

Chemically synthesized L5P (DFNMLVLILFLA) was kindly provided by Frank Biet (INRA centre de Tours, Nouzilly, France). Peptides MAP3865c_133–141_ (LAANFVVAL), MAP3865c_125-133_ (MIAVALAGL), MAP2404c_70-85_ (RGFVVLPVTRRDVTDV) and MAP1,4αgbp_157-173_ (GTVELLGGPLAHPFQPL) along with their respective human-derived homologs ZnT8_186-194_ (VAANIVLTV), ZnT8_178-186_ (MIIVSSCAV), PI_46-61_ (RGFFYTPKTRREAEDL) and PI_64-80_ (GQVELGGGPGAGSLQPL) were synthesized at 85–98% purity (LifeTein, South Plainfield, NJ 07080, USA).

### ELISA assay

Abs specific for L5P antigen, together with those directed against MAP3865c/ZnT8, MAP1,4αgbp/PI and MAP2404c/PI homologous pairs were detected in plasma samples through indirect enzyme-linked immunosorbent assays as described by Masala *et al*. [4], using final concentration of 10 μg/ml for all peptides. Each assay included a highly positive control serum for data normalization with Abs reactivity established at 1.0 arbitrary units (U/ml). Optimal positivity thresholds for the homologs were set as previously reported [16]. Cut-off value discriminating between samples positive and negative for L5P was calculated based on ROC curve and accounted for 0.83 U/ml with specificity of 83.33%.

### Statistical analysis

Statistical significance of the data was determined through the unpaired Mann-Whitney *U* test (95% CI, two-tailed) using GraphPad Prism software (version 6.02, GraphPad Software Inc., La Jolla, CA 92037, USA). Relationship between variables correlated with positivity to L5P was assessed through principal component analysis employing XLSTAT software (version 2015.1, Addinsoft, Paris, France). The variable relative to HLA-inferred genetic susceptibility was classified in three groups according to low-, moderate- and high-risk T1D haplotype.

## Results

**Fig 1:**
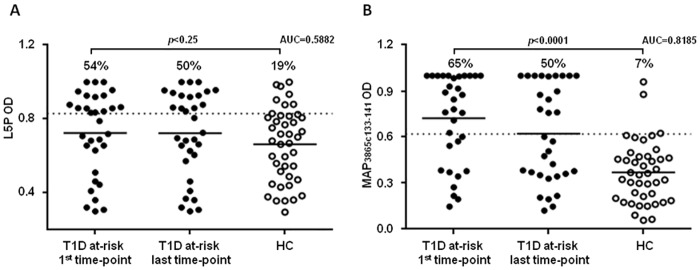
Prevalence of Abs against L5P and MAP-derived ZnT8 homolog in T1D at-risk subjects and HC. Plasma samples were analyzed in duplicate for Abs directed against L5P (A) and MAP3865c_133-141_ (B) epitopes. Distributions are relative to the sample sets including only the first or the last time-point collection in comparison to HC. Horizontal bars indicate means. The dotted line corresponds to the Abs positivity threshold. AUC and *p*-values relative to T1D at-risk subjects vs. HC test are given in the upper part. Specific percentage of reactivity is indicated above each distribution.

81.25% of children at risk for T1D and 16.67% of HC were positive to at least one of the eight homologous peptides. Reactivity to L5P antigen was lower among at-risk subjects and accounted for 54.12%, whereas responses in HC reached 19.05%. Nevertheless, the difference between means relative to L5P-positive cases and HC was not markedly pronounced and the results did not attain statistical significance (Fig 1A). MAP-derived epitopes homologous to human ZnT8 showed the highest and equal correspondence with levels of anti-L5P Abs, thus MAP3865c_133–141_ has been chosen as a representative peptide for further analysis. Similarly, our previous results demonstrated remarkable prevalence (>60%) of Abs directed against both MAP/ZnT8 homologs with high degree of statistical significance (*p*<0.0001) in comparison to values obtained for MAP/PI homologs (prevalence <23%) [16]. In this study, the initial seroreactivity to MAP3865c_133–141_ among T1D at-risk subjects was higher compared to that of L5P and reached 65% but followed a decrease to 50% when samples relative to the last blood collection were included. Prevalence among HC accounted for only 7% and the results were highly significant (*p*<0.0001; Fig 1B). Both epitopes presented a 1:1 ratio of Abs positivity between males and females. When genders were assessed separately, prevalence among girls was higher than in boys (92.3% vs. 63.7% for the homologous peptides and 69.2% vs. 42.1% for L5P).

**Fig 2:**
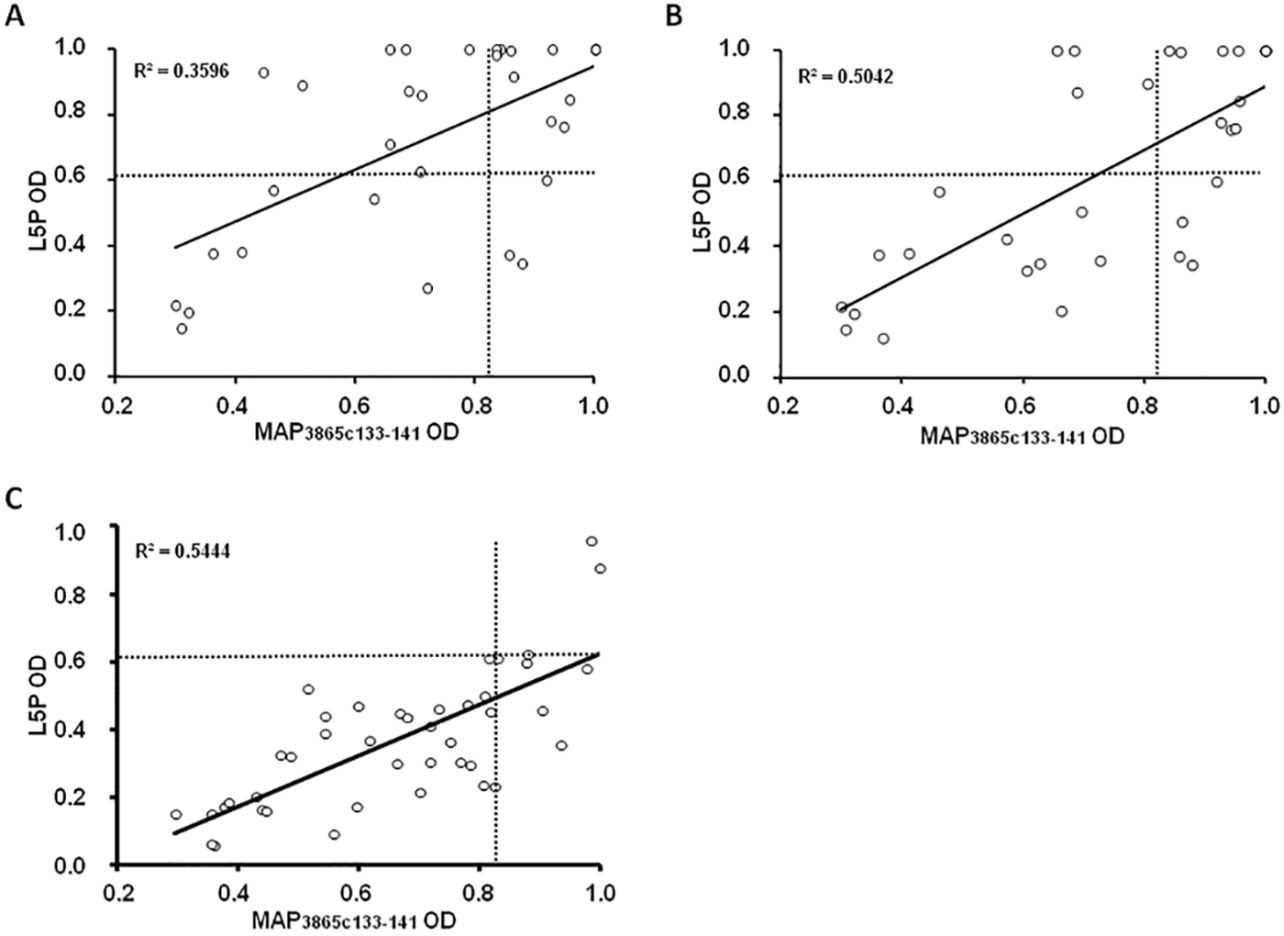
Correlation between Abs recognizing MAP-derived epitope and L5P antigen in children at risk for T1D and HC. The distributions represent correlations between Abs against MAP3865c_133–141_ and L5P in T1D at-risk subjects including samples of the first time-point (A) or the last time-point (B) and HC (C). Each circle corresponds to Abs detected in one sample. The dotted lines indicate cut-off values used in each assay to discriminate between positive and negative samples. R^2^ coefficients are given for each distribution.

Upon analysis of time-point samples, a stronger correlation between titers of Abs directed against MAP3865c_133–141_ and L5P was observed among HC and T1D at-risk subjects including values relative to the last blood collection compared to T1D at-risk children considering the first time-point samples (Fig 2). Children who maintained in time anti-MAP Abs positivity displayed a continuous response to L5P antigen, whereas loss of immunity against MAP epitopes was mirrored by reduced anti-L5P reactivity leading to a decrease of Abs levels below the established threshold. In these terms, coincidence in positivity to both MAP homologs and L5P antigen reached 90.63% in T1D at-risk subjects and 90.48% in HC, pointing at the presence of immune responses independent from cross-reactivity observed for the homologous epitopes. 7 out of 11 children for whom time-point samples were available lost their positivity to MAP-derived homologous peptides. There were no cases of time-related acquisition of either anti-MAP or anti-L5P reactivity among the selected samples (Table 1). These results reflect outcomes of the earlier study where we described a time-dependent decrease in Abs responses towards the analyzed epitopes with strikingly definite trends for MAP/ZnT8 homologs that corresponded to a similar scenario relative to standard anti-ZnT8 Abs analyzed for T1D diagnostic purposes [16].

**Table 1:** Clinical characteristics and Abs status in T1D at-risk subjects. Plus signs and hyphens indicate, respectively, the presence or absence of available time-point samples, Abs directed against MAP homologous peptides and L5P antigen with their maintenance in time, as well as coincidence of Abs status for both types of epitopes.

N.	Patient ID	Gender	Age	Time-point	Abs positivity		Abs maintenance		Coincidence
					MAP	L5P	MAP	L5P	
1	1	M	12,65	-	+	+			+
2	2	F	9,22	-	+	+			+
3	3	F	4,08	-	+	+			+
4	4	M	5,61	-	-	-			+
5	5	M	18,89	-	-	-			+
6	8	F	8,61	+	+	-	-	-	+
7	9	M	4,91	+	+	+	+	+	+
8	10	F	4,76	-	-	-			+
9	11	F	9,71	-	+	+			+
10	12	M	8,15	-	-	-			+
11	13	M	5,31	-	+	+			+
12	14	M	6,43	-	+	+			+
13	16	F	7,16	-	+	-			-
14	22	M	7,97	+	+	+	-	-	+
15	23	M	12,87	+	+	-	-	-	+
16	24	M	5,74	+	+	-	-	-	+
17	26	F	6,32	+	+	+	+	+	+
18	27	M	7,18	+	+	-	-	-	+
19	31	M	12,92	-	-	-			+
20	33	M	15,12	+	+	+	+	+	+
21	34	F	5,09	+	+	+	+	+	+
22	35	M	8,12	-	+	-			-
23	36	M	3,97	-	-	-			+
24	43	M	10,4	-	+	-			-
25	48	F	11,86	-	+	+			+
26	52	M	9,12	-	+	+			+
27	53	F	10,02	+	+	-	-	-	+
28	54	F	14,06	-	+	+			+
29	55	F	11,41	-	+	+			+
30	56	M	9,84	-	+	+			+
31	57	M	9,99	+	+	-	-	-	+
32	58	F	7,36	-	+	+			+

^a^ F: females, M: males.

^b^ Age at blood collection; relative to the first sample for subjects with multiple time-point collections.

^c^ Given only for subjects for whom time-point samples were available.

^d^ Calculated based on the Abs status against MAP and L5P epitopes for single samples or its maintenance upon time-related analysis.

**Fig 3:**
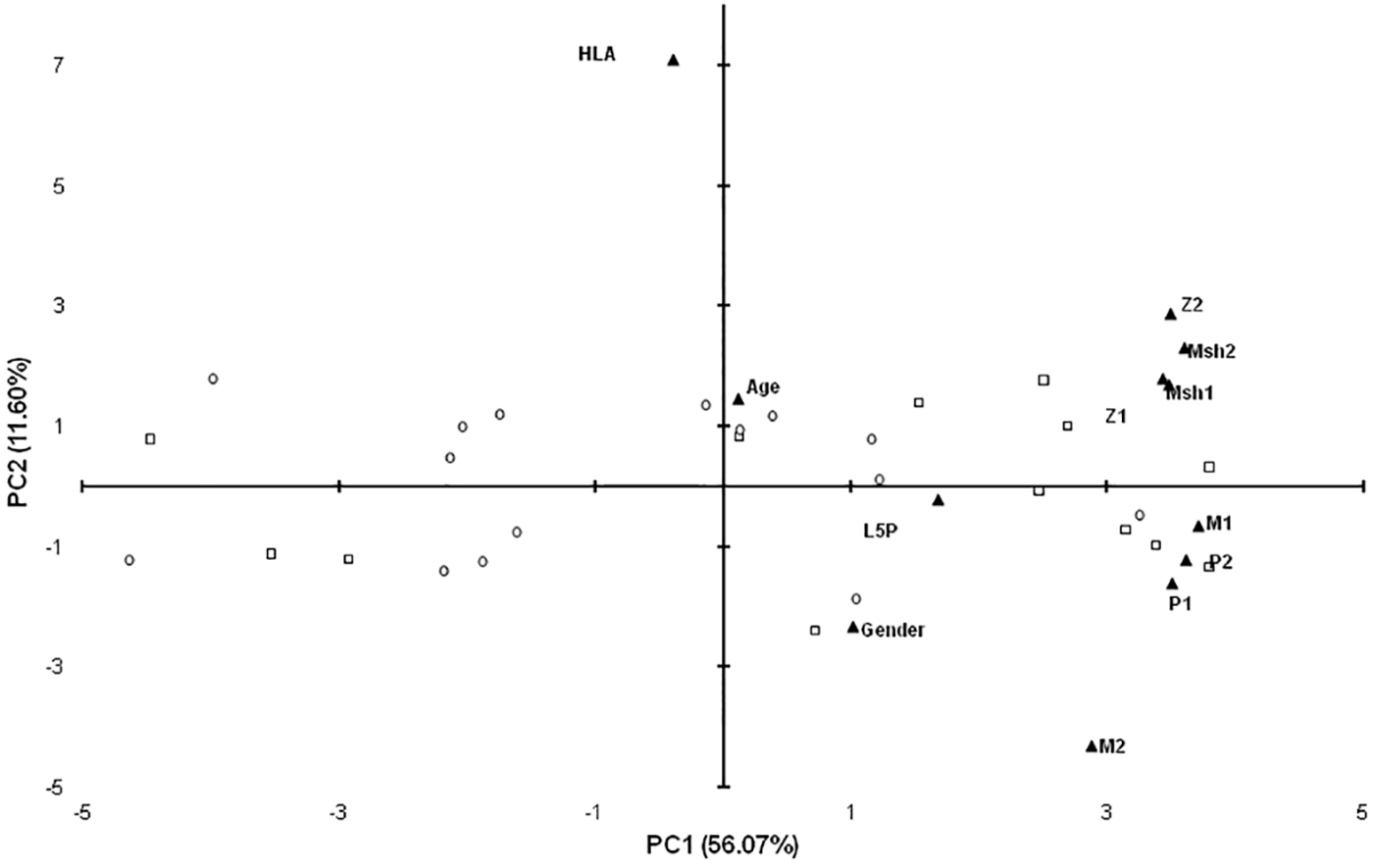
Principal component analysis of variables describing relationship with positivity to L5P in samples of children at risk for T1D. Bi-plot illustrates correlation between levels of anti-L5P Abs and variables relative to T1D genetic predisposition, available demographics data and titers of Abs against MAP-derived homologous peptides: MAP3865c_133–141_ (Msh1); MAP3865c_125-133_ (Msh2); MAP2404c_70-85_ (M1); MAP1,4αgbp_157-173_ (M2); ZnT8_186-194_ (Z1); ZnT8_178-186_ (Z2); PI_64-80_ (P1) and PI_46-61_ (P2). Children reactive to L5P antigen are indicated by squares whereas circles correspond to negative samples. All variables are described by labels and their position on the plot is indicated by triangles. The distribution shows relationship between PC1 and PC2 explaining 67.67% of the total variation. Only samples of children with known HLA genotype are included.

Principal component analysis confirmed the association of Abs levels against MAP/human homologs with L5P but revealed a weak relationship between HLA genotype and reactivity to either homologous peptides or L5P antigen (Fig 3). Among L5P-positive subjects, 46.67% had low-risk genotype, 55.55% moderate and 25% high genetic predisposition for T1D. Interestingly, in the study population, most of the high- and moderate-risk genotypes were found among males indicating an increased probability for the development of clinical symptoms by the combination of sex/genetics-related factors [19]. Age and the presence of islet cell autoantibodies were not associated with anti-L5P Abs levels or general positivity to L5P.

## Discussion

In light of significantly high responses to peptides derived from MAP3865c, MAP1,4αgbp and MAP2404c proteins and their human homologs identified within ZnT8 and PI sequences obtained previously for subjects at risk for T1D, in this study we compared seroreactivity to L5P as a specific MAP antigen in the same individuals and healthy volunteers. Considering the particular structure of L5P that lacks a free hydroxyl group in its core and presents an unmodified saturated fatty acid in the *N*-terminal domain [14], we tested several ELISA protocols, however the best sensitivity has been achieved applying the same procedure and antigen concentration as for the other epitopes. In result, anti-L5P responses were higher in children at risk for T1D than in HC but not statistically significant, in contrast to the other MAP-derived antigens. Abs prevalence to L5P and the four MAP peptides analyzed together resulted similar in HC while differed greatly in at-risk subjects (81.25% vs. 54.12%, respectively).

When initial reactivity to L5P was compared to responses against only MAP3865c_133–141_ peptide presenting the most similar positivity pattern, Abs prevalence appeared much higher in HC (19% vs. 7%, respectively) and quite lower in children at risk for T1D (54% vs. 65%, respectively). Interestingly, seroreactivity against either single MAP3865c_133–141_ or the entire group of MAP-derived and the homologous human epitopes was almost identical as that elicited by L5P antigen upon inclusion of samples relative to further time-point blood collections, resulting in the final Abs coincidence exceeding 90%. This phenomenon was due to the time-related loss of anti-MAP Abs in some children mirrored by the absence of positivity to L5P. It is plausible that MAP-derived homologs cross-react with ZnT8 and PI peptides at a young age inducing initially immune responses in subjects predisposed for T1D that are subsequently attenuated. As suggested for Crohn’s disease, Abs prevalence among healthy controls and individuals who don’t progress to overt diabetes may result from latent infection or past exposure to MAP in early childhood conferring some natural protection [20].

ZnT8 and insulin autoantibodies (IAA), together with other islet cell antigens, are standard biomarkers indicating an increased risk for T1D, especially in cases of multiple Abs positivity. Although, they cannot be detected during the first months after birth and their levels decrease with age. We previously reported that immune responses to MAP peptides appear before classical Abs used for T1D diagnosis [21], however in many cases they tend to attenuate. In this context, L5P might have a general diagnostic potential but, in the analyzed samples, it was not sensitive to changes in seroreactivity against MAP observed for the other epitopes. Nonetheless, positivity to L5P in HC corresponded to values below the cut-off point obtained for MAP-derived and human homologous peptides, and was reflected by a significant correlation performed for MAP3865c_133–141_. On the other hand, L5P may be helpful to immediately indicate the presence of anti-MAP Abs when evaluation of a more complex Abs status, achievable by the application of the homologous epitopes, is not required, avoiding false-positive results due to temporal cross-reactivity.

This study provides evidences that either protein or lipid MAP antigens may induce similar Abs responses in terms of strength, while somewhat higher specificity corresponding to the optimal positivity threshold characterized the selected MAP peptides. Since L5P has been described as more specific than the current diagnostic test for Johne’s disease based on MAP purified protein derivative (PPD) [14], almost complete coincidence in responses to both types of antigens used in the present study lends support to the involvement of anti-MAP Abs in T1D. These responses should be further assessed with regard to cell-mediated immunity. In fact, during early stages of MAP infection, cattle and sheep typically display a Th1-dominant condition subsequently switched to a Th2 response [22]. Similar shift has been observed in NOD mice and T1D at-risk subjects, followed by disappearance of T-cell responses after clinical onset [23]. Yet, cytokine production pattern involving IL-17, IL-22 and INFγ is common to individuals affected by autoimmune diabetes and those with mycobacterial infections [24, 25, 26, 27]. Other studies reported a significantly increased *in vitro* secretion of IL-1β, IL-6, IL-8, IL-10 and TNF-α in peripheral blood mononuclear cells of diabetic patients treated with mycobacterial Hsp65 protein or synthetic peptides corresponding to portions of MAP-derived Hsp65 [28]. On the contrary, the same group reported no significant seroreactivity against MAP3738c epitope in T1D cohort from Indian Hyderabad area [29] previously detected in Sardinian T1D population [30]. These differences highlight the diversity of responses induced by various MAP antigens in distinct biogeographical backgrounds, thus exploring new targets may help to unravel the elusive mechanism through which MAP could be involved in the pathogenesis of T1D with respect to predisposing genetic polymorphisms.

Even though our results did not show statistical significance when immunoreactivity against L5P in children at risk for T1D was compared to L5P Abs prevalence among HC, they showed almost complete coincidence with other anti-MAP responses characterized by a highly significant *p*-value. L5P time-related antigenicity needs further evaluation in a larger group of children at risk for T1D, with ensuing follow up studies including younger subjects. Assessment of anti-L5P Abs at T1D onset and in adults with established autoimmune diabetes would complement our previous results with the degree of humoral response stability. Efforts to understand the pathogenicity of MAP in humans would shed light on its zoonotic potential described in connection with various autoimmune diseases [31]. Higher prevalence of Abs against L5P and MAP-derived peptides in subjects with low and moderate T1D risk conferred by HLA haplotypes is in line with the current onset trends in less genetically predisposed individuals [32] and makes MAP a conceivable environmental agent at play in T1D development.

## Abbreviations

Abs: antibodies
GPLs: glycopeptidolipids
HC: healthy controls
IAA: insulin autoantibodies
L5P: lipopentapeptide
MAP: *Mycobacterium avium* subsp. *paratuberculosis*
PI: proinsulin
PPD: purified protein derivative
ROC: receiver operating characteristic
T1D: type 1 diabetes
ZnT8: zinc transporter 8 protein
