# Correction: Seroreactivity against Specific L5P Antigen from *Mycobacterium avium* subsp. *paratuberculosis* in Children at Risk for T1D

**DOI:** 10.1371/journal.pone.0161516

**Published:** 2016-08-16

**Authors:** Magdalena Niegowska, Novella Rapini, Frank Biet, Simona Piccinini, Sylvie Bay, Roberta Lidano, Maria Luisa Manca Bitti, Leonardo A. Sechi

There is an error in the first sentence of the MAP antigens section of the Methods: The sentence "Chemically synthesized L5P (DFNMLVLILFLA) was kindly provided by Frank Biet (INRA centre de Tours, Nouzilly, France)." should be replaced by "Synthetic L5P (C19H39CO-D-Phe-L-*N*-Me-Val-L-Ile-L-Phe-L-Ala-OMe) was prepared as previously described [14]."

The following information is missing from the Acknowledgements Section: The authors thank Christelle Ganneau for participating in the L5P synthesis.
